# Evaluation of Donor and Recipient Characteristics Involved in Descemet Stripping Automated Endothelial Keratoplasty Outcomes

**DOI:** 10.3389/fmed.2021.605160

**Published:** 2021-04-12

**Authors:** Michele Lanza, Rosa Boccia, Adriano Ruggiero, Paolo Melillo, Mario Bifani Sconocchia, Francesca Simonelli, Sandro Sbordone

**Affiliations:** Multidisciplinary Department of Medical, Surgical, and Dental Sciences, University of Campania Luigi Vanvitelli, Naples, Italy

**Keywords:** corneal transplantation, DSAEK, DSAEK complications, Fuch's dystrophy, bullous keratopathy

## Abstract

**Aims:** To evaluate both donor and recipient features involved in visual acuity restoring and complication insurgence in eyes that have undergone Descemet stripping automated endothelial keratoplasty (DSAEK).

**Methods:** In this retrospective study, charts of 111 eyes of 96 patients (mean age 70.25 ± 8.58 years) that underwent DSAEK were evaluated. Only Fuch's Distrophy (FD) or Bullous Keratopathy (BK) due to cataract surgery eyes were included. A complete ophthalmic check with endothelial cell density (ECD) and central corneal thickness (CCT) measurement was performed before surgery and at 1, 3, 6, and 12 months follow-up. Each DSAEK was performed by the same well-trained surgeon; only pre-cut lenticules, provided by same Eye Bank, were implanted.

**Results:** A total of 48 (43%) complications have been observed (most of them were 22 partial graft detachments and 17 IOP spikes). At the last follow-up (mean: 8.58 ± 4.09 months), a significant increase (*p* < 0.05) of best corrected visual acuity (BCVA) was detected. Overall mean BCVA of the eyes evaluated was 0.40 ± 0.43 LogMAR with BK eyes showing a significantly higher improvement (*p* < 0.05) compared to FD eyes. The only factor showing a significant correlation (*p* < 0.05) with visual acuity enhancement was the implant of a lenticule thinner than 100 μm. Recipient features significantly (*p* < 0.05) associated with complications observed after surgery were glaucoma and diabetes mellitus.

**Conclusion:** The use of a graft thinner than 100 μm can provide better visual acuity recovery while recipients affected by glaucoma or diabetes mellitus are more prone to develop complications after surgery.

## Introduction

The evolution of corneal transplantation techniques has led to the development of endothelial keratoplasty (EK) procedures such as Descemet stripping automated endothelial keratoplasty (DSAEK) and Descemet membrane endothelial keratoplasty (DMEK) that, today, are gradually becoming the preferred surgical choice to treat diseases involving the endothelial layer such as Fuchs' dystrophy (FD) and bullous keratopathy (BK) (1–4). Descemet stripping automated endothelial keratoplasty, a surgical procedure aiming to replace endothelium and Descemet membrane with donor tissue composed by endothelium, Descemet membrane and posterior stroma and it is the most commonly performed endothelial keratoplasty, whereas during DMEK surgery, the endothelial layer is replaced by a lenticule thinner than 15 μm, free of deep stroma, this last technique is described to have a quicker vision recovery with an higher incidence of complications (3–5). Currently there is a lack of unanimous superiority of one EK technique over the other, also due to the continuous evolution of both (4–6).

Even if penetrating keratoplasty (PK) provides comparable, long term, improvement of visual acuity, DSAEK is generally preferred because of faster visual recovery, less induced astigmatism, less suture related issues, lower rate of both rejections and wound related problems (1, 2).

Although DSAEK is commonly preferred over PK, this technique has some potential drawbacks and sometimes eyes having this type of surgery show a poor visual acuity improvement even without complications (6, 7). For this reason, it is important to analyse the features characterizing DSAEK surgery in order to identify the factors potentially correlated with complication insurgence and good visual acuity recovery. Some multicentre studies analyzing these factors have been published (3, 8–10), but the study described here evaluates a standardized technique performed by a single well-trained surgeon (SS), erasing bias related to the different expertise of performing surgeons. Moreover, this is one of the first studies that reports the influence of donor and recipient features not only regarding complication onset but also on visual acuity restoring.

## Materials and Methods

This study included 111 eyes of 96 patients ranging from 39 to 88 years old (mean age: 70.25 ± 8.58 years), referring to the Ophthalmology Unit of the Multidisciplinary Department of Medical, Surgical and Dental Sciences of the University of Campania “Luigi Vanvitelli” that underwent DSAEK between February 2012 and 2020. Patient charts have been revised and analyzed. Inclusion criteria were adult patients with irreversible corneal endothelial dysfunction due to endothelial decompensation after cataract surgery or due to Fuchs' dystrophy requiring corneal transplantation with no coexisting vision-limiting comorbidities other than cataract. Patients affected by corneal decompensation due to different causes, such as eyes with graft rejection or previous corneal transplant surgery, eyes requiring secondary scleral or iris fixated IOL implant, anterior or posterior vitrectomy, nucleus removal from vitreous cavity, or other causes of endothelial decompensation were excluded from the study to eliminate bias on the overall outcome analysis.

Patient evaluations were performed both before and after surgery. Post-surgery evaluations were performed at day 1, after 1 week and after 1, 3, 6, and 12 months. Each surgical procedure was performed by the same well-trained surgeon (SS).

Diseases and ocular details of the eyes included in the study are summarized in Table 1. The most frequent systemic diseases affecting patients enrolled in this study were systemic hypertension, detected in 73 patients (79.17%), and diabetes mellitus (DM), observed in 26 patients (23.42%); main ocular comorbidities were compensated glaucoma with no worsening of visual field in last 2 years, detected in 14 eyes (12.61%), and non-proliferative diabetic retinopathy, observed in 7 eyes (6.3%).

**Table 1:** Baseline demographic, and ocular characteristics of the sample analyzed.

	**Mean ± SD (range)**
Age (years)	70.25 ± 8.58 (from 39 to 88 old)
	Number
Male	45 (45.78%)
Right eye	57 (51.31%)
**Diagnosis (*****n*****)**	
- Fuchs dystrophy	55
- Bullous keratopathy	56
**Lens status (*****n*****)**	
- Clear lens	3
- Cataract	52
- Pseudophakic	56
**Type of surgery**	
- DSAEK	57
- Triple procedure	54
**Visual acuity**	**Mean** **±** **SD (range)**
• Features	
• UCVA (LogMAR)	1.45 ± 0.59 (from 2.77 to 0.3)
• BCVA (LogMAR)	1.08 ± 0.69 (from 2.77 to 0)
**Refraction**	
- Sphere (*D*)	0.24 ± 2.96 (from −16 to + 5)
- Cylinder (*D*)	0.15 ± 1.56 (from −3.25 to +3.5)
- Spherical equivalent (*D*)	0.31 ± 3.24 (from −16 to +6.125)
• CCT (μm)	630.56 ± 146.59 (from 455 to 993)

*DSAEK, Descemet stripping automated endothelial keratoplasty; UCVA, uncorrected visual acuity; BCVA, best-corrected visual acuity; CCT, central corneal thickness*.

Data acquired during visits before surgery and at 1, 3, 6, and 12 months follow up were included in the statistical evaluation. The patients underwent a complete ophthalmic examination including uncorrected visual acuity (UCVA) and best-corrected visual acuity (BCVA), measured as Snellen lines and converted to LogMAR, refraction evaluation, slit lamp exam, intraocular pressure (IOP) measured by Goldmann applanation tonometry, corneal endothelial cell density (ECD) evaluation and central corneal thickness (CCT) assessment, using EM-3000 Specular Microscope (Tomey Corporation, Nagoya, Japan). Patients undergoing phacoemulsification with intraocular lens (IOL) implantation and DSAEK (triple procedure), underwent both axial length, corneal curvature measurements and intraocular lens (IOL) power calculation using IOLMaster 500 (Carl Zeiss, Jena, Germany) and SRK/T formula aiming to reach−1 D refraction.

Donor corneal lenticules were provided by a unique Eye Bank: Eye Bank of Mestre (Italy), they were pre-cut and preserved according to conventional eye-bank techniques. Details about donor graft such as thickness, ECD, time from death to preservation and time of preservation are shown in Table 2.

**Table 2:** Details of graft used in this study.

	**Mean (SD) or *n* (%)**	**Minimum**	**Maximum**
Age of donor (years)	62.02 ± 10.51	22	80
LT (μm)	90.27 ± 20.87	45	163
ECD (cell/mm^2^)	2593.64 ± 116.74	2,300	2,900
DPT (hours)	18.63 ± 12.52	2.35	93.5
PT (day)	16.27 ± 4.33	10	30

*LT, lenticule thickness; ECD, endothelial cell density; DPT, death to preservation time: time from death to preservation in storage solution; PT, preservation time: time from preservation and surgery*.

Two kinds of anesthesia were used, peri-bulbar anesthesia was adopted in 105 eyes, 52 BK eyes, and 53 FD ones, while a sub-tenonian procedure was chosen in 6 BK eyes and 2 FD ones.

Peribulbar anesthesia was performed using 9 ml of 0.75% ropivacaine combined with 100 IU hyaluronidase; subtenonian anesthesia was performed using 1 ml of 0.75% ropivacaine.

Descemet stripping automated endothelial keratoplasty procedures were performed with the surgeon sitting at the temporal position, according to the standard technique previously described (11), except for the following modifications:

- in patients who underwent DSAEK, descemetorhexis was conducted under BSS after anterior chamber maintainer insertion;
- in patients who underwent triple procedure, after IOL injection, the surgeon performed descemetorhexis under viscoelastic material, 1% sodium hyaluronate (Alcon, Forth Worth, USA).

In eyes requiring triple procedure, phacoemulsification with chop *in situ* technique (12) and IOL implant was performed before DSAEK through a 2.75 mm corneal tunnel opening into the anterior chamber. This tunnel was later enlarged to 4 mm for the insertion of the donor tissue. Wounds were secured with 10/0 nylon interrupted sutures, and a large air bubble in the anterior chamber was gently injected to allow the graft adhesion to the recipient stroma. Subconjunctival injection of 1 ml of 4% gentamicin plus 1 ml of 0.2% betamethasone and a sterile bandage completed the procedure. The patient was prescribed to lay in a supine position for at least 3 h. IOP was monitored post-operatively every 30 min using TAO1i rebound tonometer (ICARE, Vantaa, Finland), to avoid pupil block damage. If a value higher than 30 mmHg was found during the first 24 h, a partial evacuation of the anterior chamber air bubble was performed at slit lamp.

After surgery, each patient received a topical therapy with a fixed association of 3 mg/mL netilmicin and 1 mg/mL dexamethasone eye drops 4 times daily for the first 30 days. Thereafter, post-operative treatment included the instillation of topical 1 mg/dL dexamethasone 3 times a day for 1 month, then tapered to 2 times daily for 2 more months and finally to 1 drop daily for the last 2 months.

The increase in BCVA after surgery was considered as the primary outcome, whereas the absence of complications was considered a secondary one.

This study evaluated the correlations among the BCVA improvement observed at the last follow up and features such as endothelial disease (FD vs. BK), donor characteristics, general and systemic comorbidity of the recipient. Moreover, the correlation of these factors with the insurgence of complications during or after surgery have been studied, in order to detect those involved in a successful DSAEK and the features that could instead increase the complication rate.

### Statistical Analysis

Continuous variables are reported as mean ± standard error of the mean (SEM) and categorical variables are reported as counts (frequency).

Linear regression, estimated by a generalized estimating equation (GEE), was fitted on the data of the last visit compared to the baseline visit to estimate the change of each outcome (i.e., BCVA, CCT, and EDC loss), also in relationship with selected factors (e.g., diseases, surgical techniques, etc.). Logistic regression, estimated by GEE, was fitted to explore the relationship between the selected binary outcome (i.e., complications and rebubbling) and the selected factor. GEE was applied since this method could accommodate the inter-eye correlation (i.e., between the 2 treated eyes of the same subject) by adopting an appropriate covariance structure (13), also in case of non-normality of the data (14).

## Results

At the last follow up, mean: 8.58 ± 4.09 months (from 1 to 12 months), a significant increase (*p* < 0.01) of BCVA was observed in the overall sample, reaching a mean value of 0.40 ± 0.43 LogMAR (ranging from 0.00 to 2.10). At the same follow up, mean CCT was 572.49 ± 49.64 μm (ranging from 481 to 725 μm) and mean ECD was 1955.92 ± 301.54 cells/mm^2^ (ranging from 1,147 to 2,674 cells/mm^2^).

**Figure 1:**
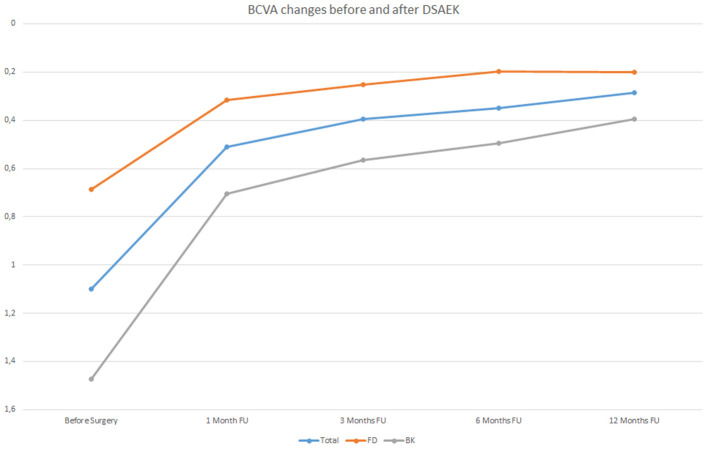
Best Correct Visual Acuity (BCVA) changes in LogMAR before Descemet stripping automated endothelial keratoplasty (DSAEK) at 1, 3, 6, and 12 months follow-up (FU) both in the overall study population (blue line) and in Fuchs dystrophy (FD) eyes (orange line) and in bullous keratopathy (BK) eyes (gray line).

The increase of BCVA in the whole sample evaluated and both in FD and BK eyes is graphically represented in Figure 1.

As can be observed in Table 3, BK eyes showed a significantly higher visual acuity enhancement (*p* < 0.01) compared to FD eyes after DSAEK. It is important to highlight that, before surgery, BCVA values of BK eyes (mean: 1.47 ± 0.64 LogMAR) were significantly lower (*p* < 0.05) than those of FD eyes (mean: 0.69 ± 0.49 LogMAR).

**Table 3:** Influence of recipient's factors on BCVA improvement, CCT variation, and ECD loss observed after surgery, measured at last follow-up (mean: 8.58 ± 4.09 months, from 1 to 12 months).

	**Mean BCVA improvement**	***p*-value**	**% of CCT loss**	***p*-value**	**% of ECD loss**	***p*-value**
Overall cohort	0.68 ± 0.59	<0.001	1.7 ± 7.1	0.087	24.0 ± 11.7	<0.001
Fuchs dystrophy	0.47 ± 0.46	<0.001	2.3 ± 5.8	0.477	24.4 ± 12.2	0.830
Bullous keratopathy	0.89 ± 0.64		0.9 ± 8.5		23.6 ± 11.3	
Diabetes mellitus	0.87 ± 0.65	0.142	3.7 ± 8.4	0.156	25.6 ± 13.3	0.479
Systemic hypertension	0.74 ± 0.62	0.160	1.3 ± 7.9	0.602	24.3 ± 12.5	0.697
Glaucoma	0.46 ± 0.67	0.265	4.0 ± 11.4	0.390	23.8 ± 15.2	0.998
Diabetic retinopathy	0.64 ± 0.55	0.736	2.1 ± 8.1	0.898	0.19 ± 11.4	0.339
DSAEK	0.87 ± 0.64	<0.001	0.8 ± 8.5	0.477	23.5 ± 11.2	0.615
Triple procedure	0.49 ± 0.46		2.3 ± 5.7		24.5 ± 12.3	
Overall complications	0.78 ± 0.60	0.160	2.0 ± 8.4	0.583	25.2 ± 12.9	0.425
Elevation of IOP	0.80 ± 0.59	0.342	0.7 ± 8.3	0.491	25.6 ± 13.3	0.407
Rebubbling	0.61 ± 0.59	0.485	0.6 ± 7.7	0.485	23.7 ± 14.1	0.867

*DSAEK, Descemet stripping automated endothelial keratoplasty; LT, lenticule thickness; DPT, death to preservation time; PT, preservation time; IOP, intraocular pressure*.

**Figure 2:**
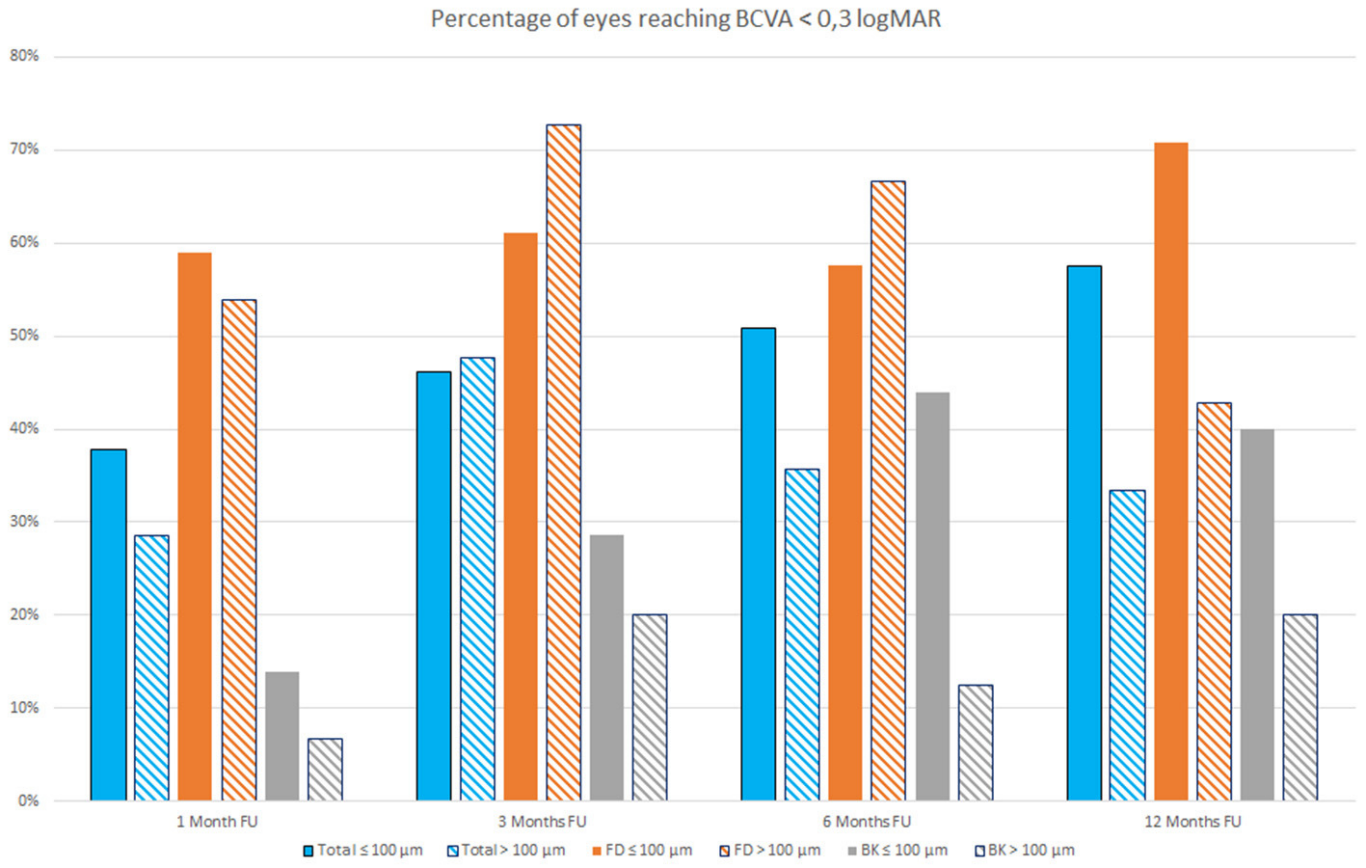
Percentage of eyes reaching Best Correct Visual Acuity (BCVA) <0.3 LogMAR at 1, 3, 6, and 12 months follow-up (FU) that received a lenticule thinner (full colored bars) or thicker than 100 μm (stripes colored bars) both in the overall study population (blue bars) and in Fuchs dystrophy (FD) eyes (orange bars) and in bullous keratopathy (BK) eyes (gray bars).

Figure 2 shows the percentages of eyes reaching a BCVA lower than 0.3 logMAR at 1, 3, 6, and 12 months follow up in eyes that received a lenticule thinner or thicker than 100 μm; the bars represent both the overall sample evaluated and the FD and BK eyes.

It is possible to observe that during first months after surgery, there are little differences in the percentage of eyes reaching a BCVA lower than 0.3 logMAR among patients with FD that received a lenticule thinner or thicker than 100 μm, whereas this difference became more evident at 12 months follow up (Figure 2).

The 48 complications observed in the sample analyzed are reported in Table 4. Partial lenticule detachments were observed early after surgery and required additional maneuvers to restore the graft position, IOP spikes (>30 mmHg) were reported both early and later during the follow up; among others, 1 graft rejection, 1 blood detected into interface, 1 persistent epithelial defect, and 1 suture dehiscence were reported.

**Table 4:** Post-operative complications.

	***n***	**%**
Partial graft detachment	22	19.82
IOP spikes	17	15.31
PCO	3	5.77
CME	2	1.80
Others	4	3.60

*IOP, intraocular pressure; PCO, posterior capsule opacification; CME, Cystoid macular oedema*.

During follow up, no significant CCT changes were observed, whereas a significant (*p* < 0.05) loss of mean 24% of ECD was observed, with no differences between FD and BK eyes (Table 4).

According to the regression models corrected for the diseases shown in Table 5, the implant of a graft thickness lower than 100 μm was the only factor that significantly influenced BCVA improvement. No other donor features, such as age, ECD of the graft, death to preservation time or the preservation time showed a significant correlation with visual acuity enhancement.

**Table 5:** Analysis of both recipient- and donor-related factors on BCVA improvement by regression models corrected for disease.

**Features**	***p*-value**
Diabetes mellitus	0.229
Systemic hypertension	0.204
Glaucoma	0.105
Diabetic retinopathy	0.401
DSAEK	0.788
LT < 100 μm	<0.05
DPT > 10 h	0.526
PT > 14 days	0.740
Overall complications	0.145
Elevation of IOP	0.507
Rebubbling	0.125

*DSAEK, Descemet stripping automated endothelial keratoplasty; LT, lenticule thickness; DPT, death to preservation time; PT, preservation time; IOP, intraocular pressure*.

DM and Glaucoma appeared to be the only factors that showed a significant correlation with the insurgence of complications (Table 6).

**Table 6:** Correlation between post-operative complications and systemic, ocular and graft features at baseline.

	**With complication (*n*. 48)**	**Without complication (*n*. 63)**	***p*-value**
Fuchs dystrophy	23 (47.9%)	25 (39.7%)	0.617
Diabetes mellitus	16 (33.3%)	10 (15.9%)	<0.05
Systemic hypertension	33 (68.7%)	40 (63.5%)	0.551
Glaucoma	10 (20.8%)	4 (6.3%)	<0.05
Diabetic retinopathy	4 (8.3%)	3 (4.8%)	0.426
LT > 100 μm	8 (16.7%)	19 (30.2%)	0.129
DPT > 10 h	34 (70.8%)	41 (65.1%)	0.492
PT > 14 days	30 (62.5%)	28 (44.4%)	0.163

*LT, lenticule thickness; DPT, death to preservation time; PT, preservation time*.

## Discussion

The introduction of endothelial keratoplasty (EK) revolutionized corneal transplantation in the last years (3, 10). In the United States and in Europe, DSAEK replaced PK as the most performed corneal transplantation technique, while Fuchs' endothelial dystrophy has become the most common indication for this kind of procedure (3, 10). This technique produced excellent results and technique's variations aiming to improve the efficacy of DSAEK are continuously proposed (3, 14). In particular, Busin et al. (15) firstly introduced the use of an ultrathin lenticule in 2012, <100 μm in thickness, with very good results and it is now a diffusely accepted procedure among DSAEK surgeons.

In order to improve the results of this technique, it is important to detect the characteristics correlated to better results but also to be able to identify factors that could cause complications or compromise good visual acuity restoring.

In this study, partial graft dislocation was the most frequent complication observed (22 eyes) and has been always managed by early rebubbling.

Studies carried out by the Cornea Preservation Time Study (CPTS) group provided important efficacy and safety data on DSAEK technique (8, 16–20). These multicentre, randomized trials evaluated the incidence of several factors on graft failure. The sample analyzed is much larger compared to the population of this study but some of their findings are in agreement with our data: graft failure is a rare event, diabetes and glaucoma play a significant role in graft related complications compared to lenticule preservation time or endothelial cell loss after surgery. These studies focused more on graft related complications and failure, whereas this study aims to evaluate both donor and recipient factors, involved in BCVA gain after DSAEK together with those associated with complications.

The data observed in present study suggest that graft thickness <100 μm provides better visual acuity (Table 5), whereas other thickness ranges were not associated to a significant BCVA improvement. This is an important finding because the debate about the ideal thickness of the DSAEK graft, able to provide better results, is still open (3, 15).

An accurate evaluation of study data shows that, at 12 months follow up, both overall sample and FD and BK eyes showed a higher percentage of eyes with a BCVA lower than 0.3 logMAR when a lenticule with a thickness ≤ 100 μm was implanted, even if FD eyes that received a lenticule thicker than 100 μm showed better results during the first months after surgery. It is important to take into account that this behavior could, however, differ evaluating larger samples.

Thus, this study supports the results provided by Madi et al. (21) on the efficacy of ultrathin DSAEK even if, it is important to evaluate these cases in a follow up longer than 6 months. Moreover, this study highlights the importance of donor characteristics such as age, ECD of the graft, death to preservation time or lenticule preservation time. This kind of analysis is often missing in studies published on DSAEK outcomes.

The results observed here agree with those published by Suh et al. (22) as regards the rate of graft dislocations and complications but also includes a deep analysis on donor and lenticule characteristics.

Eyes with higher risk of developing graft failure such as a history of corneal viral infections, PK failures, glaucoma surgery (both trabeculectomy or tube device implants) have been excluded to avoid bias in the statistical analysis of both donor and recipient characteristics involved in a good visual acuity restoration.

BK and FD eyes have been included in this study because the surgical procedure is the same (3), while eyes needing too many additional maneuvers such as vitrectomy (both anterior and posterior) or scleral fixation IOL implant, have been excluded to eliminate bias in the statistical analysis.

The limits of this study are its retrospective design and the limited number of analyzed cases compared to papers previously published on this topic. Moreover, while some of the results shown have already been mentioned in previously published papers (21, 22) the new findings need to be confirmed in further, independent studies in order to be largely adopted by physicians. In particular, papers evaluating DSAEK almost never focused their attention on BCVA results whereas in this study a complete BCVA analysis has been provided. Furthermore, most of the published studies on this topic are multi-centric and this could introduce several biases, for example, surgeon expertise whereas, only one very well-trained surgeon performed each DSAEK in this study.

Even if this study has some limitations, it provides one of the most complete evaluations of all factors, both recipient and donor related, involved in DSAEK surgery.

The information provided here could be useful for physicians to better select which endothelial procedure to adopt in their cases. Even if DSAEK is a very diffuse technique, a debate about the superiority over DMEK is still open and further studies with more standardized endpoints are needed in order to better compare the results of both procedures.

Results observed in this study confirm that early graft dislocation is still an unsolved problem with no answer regarding onset mechanisms, but the relatively simple management explains one of the reasons why DSAEK has become very popular among cornea specialists.

In conclusion, even if further studies are needed to confirm the data observed in this evaluation, lenticules thinner than 100 μm appear to provide better results in BK and FD eyes undergoing DSAEK. The two groups of diseased eyes evaluated showed no differences in endothelial cell loss. Moreover, DM and glaucoma appear to be the only recipient diseases correlated to the insurgence of complications insurgence after surgery, thus more attention needs to be paid in shortening the follow up and recommending patients to strictly follow therapy and advices prescribed by surgeons after DSAEK.

## Data Availability Statement

The raw data supporting the conclusions of this article will be made available by the authors, without undue reservation.

## Ethics Statement

The studies involving human participants were reviewed and approved by Comitato Etico Università degli Studi della Campania Luigi Vanvitelli—Azienda Ospedaliera Universitaria Luigi Vanvitelli—AORN Ospedali dei Colli. The patients/participants provided their written informed consent to participate in this study.

## Author Contributions

FS, ML, SS, MB, and RB were responsible for the initial plan and study design. PM, RB, and AR collected and extracted data. PM and AR were responsible for statistical analysis. ML and RB interpreted data and drafted the manuscript. FS, ML, SS, and MB did a critical revision of the manuscript. All authors contributed to the article and approved the submitted version.

## Conflict of Interest

The authors declare that the research was conducted in the absence of any commercial or financial relationships that could be construed as a potential conflict of interest.

## References

[1] Ang M Soh Y Htoon HM Mehta JS Tan D. Five-year graft survival comparing Descemet stripping automated endothelial keratoplasty and penetrating keratoplasty. Ophthalmology. (2016) 123:1646–52. 10.1016/j.ophtha.2016.04.049 27262764

[2] Lee WB Jacobs DS Musch DC Kaufman SC Reinhart WJ Shtein RM. Descemet's stripping endothelial keratoplasty: safety and outcomes: a report by the American Academy of Ophthalmology. Ophthalmology. (2009) 116:1818–30. 10.1016/j.ophtha.2009.06.021 19643492

[3] Durrani AF Faith SC Jhanji V. Ultrathin descemet stripping automated endothelial keratoplasty. Curr Opin Ophthalmol. (2019) 30:264–70. 10.1097/ICU.0000000000000575 31033735

[4] Dunker SL Dickman MM Wisse RPL Nobacht S Wijdh RH Bartels MC. Descemet membrane endothelial keratoplasty vs. ultrathin Descemet stripping automated endothelial keratoplasty: a multicenter randomized controlled clinical trial. Ophthalmology. (2020)127:1152–9. 10.1016/j.ophtha.2020.02.029 32386811

[5] Chamberlain W Lin CC Austin A Schubach N Clover J McLeod SD. Descemet endothelial thickness comparison trial: a randomized trial comparing ultrathin Descemet stripping automated endothelial keratoplasty with Descemet membrane endothelial keratoplasty. Ophthalmology. (2019) 126:19–26. 10.1016/j.ophtha.2018.05.019 29945801

[6] Li JY Terry MA Goshe J Davis-Boozer D Shamie N. Three-year visual acuity outcomes after Descemet's stripping automated endothelial keratoplasty. Ophthalmology. (2012) 119:1126–9. 10.1016/j.ophtha.2011.12.037 22364863

[7] Wacker K Baratz KH Maguire LJ McLaren JW Patel SV. Descemet stripping endothelial keratoplasty for Fuchs' endothelial corneal dystrophy: five-year results of a prospective study. Ophthalmology. (2016) 123:154–60. 10.1016/j.ophtha.2015.09.023 26481820

[8] Aldave AJ Terry MA Szczotka-Flynn LB Liang W Ayala AR Maguire MG. Effect of graft attachment status and intraocular pressure on Descemet stripping automated endothelial keratoplasty outcomes in the cornea preservation time study. Am J Ophthalmol. (2019) 203:78–88. 10.1016/j.ajo.2019.02.029 30849341 PMC6612575

[9] Dickman MM Kruit PJ Remeijer L van Rooij J Van der Lelij A Wijdh RH. A Randomized multicenter clinical trial of ultrathin Descemet stripping automated endothelial keratoplasty (DSAEK) versus DSAEK. Ophthalmology. (2016) 123:2276–84. 10.1016/j.ophtha.2016.07.036 27659544

[10] Dickman MM Peeters JM van den Biggelaar FJ Ambergen TA van Dongen MC Kruit PJ. Changing practice patterns and long-term outcomes of endothelial versus penetrating keratoplasty: a prospective Dutch registry study. Am J Ophthalmol. (2016) 170:133–42. 10.1016/j.ajo.2016.07.024 27497603

[11] Busin M Madi S Santorum P Scorcia V Beltz J. Ultrathin Descemet's stripping automated endothelial keratoplasty with the microkeratome double-pass technique: two-year outcomes. Ophthalmology. (2013) 120:1186–94. 10.1016/j.ophtha.2012.11.030 23466268

[12] Vasavada AR Desai JP. Stop, chop, chop, and stuff. J Cataract Refr Surg. (1996) 22:526–52. 10.1016/s0886-3350(96)80003-4 8784620

[13] Zeger SL Liang K-Y Albert PS. Models for longitudinal data: a generalized estimating equation approach. Biometrics. (1988) 44:1049–60. 3233245

[14] Islam MA Chowdhury RI. Generalized estimating equation. In: Springer, editor. Analysis of Repeated Measures Data. Springer Singapore, FL: Springer Nature (2011). p. 161–7.

[15] Busin M Patel AK Scorcia V Ponzin D. Microkeratome-assisted preparation of ultrathin grafts for descemet stripping automated endothelial keratoplasty. Invest Ophthalmol Vis Sci. (2012) 53:521–4. 10.1167/iovs.11-7753 22205600

[16] Patel SV Lass JH Benetz BA Szczotka-Flynn LB Cohen NJ Ayala AR. Cornea Preservation Time Study Group. Postoperative endothelial cell density is associated with late endothelial graft failure after Descemet Stripping Automated Endothelial Keratoplasty. Ophthalmology. (2019) 126:1076–83. 10.1016/j.ophtha.2019.02.011 30790587 PMC6646077

[17] Lass JH Benetz BA Patel SV Szczotka-Flynn LB O'Brien R Ayala AR. Cornea Preservation Time Study Group. Donor, recipient, and operative factors associated with increased endothelial cell loss in the Cornea Preservation Time Study. JAMA Ophthalmol. (2019) 137:185–93. 10.1001/jamaophthalmol.2018.5669 30422157 PMC6439830

[18] Stulting RD Lass JH Terry MA Benetz BA Cohen NJ Ayala AR. Cornea Preservation Time Study Group. Factors associated with graft rejection in the Cornea Preservation Time Study. Am J Ophthalmol. (2018) 196:197–207. 10.1016/j.ajo.2018.10.005 30308200 PMC6258266

[19] Terry MA Aldave AJ Szczotka-Flynn LB Liang W Ayala AR Maguire MG. Cornea Preservation Time Study Group. Donor, recipient, and operative factors associated with graft success in the Cornea Preservation Time Study. Ophthalmology. (2018) 125:1700–9. 10.1016/j.ophtha.2018.08.002 30098353 PMC6196643

[20] Lass JH Benetz BA Verdier DD Szczotka-Flynn LB Ayala AR Liang W. Cornea Preservation Time Study Group. Corneal endothelial cell loss 3 years after successful Descemet stripping automated endothelial keratoplasty in the Cornea Preservation Time Study: a Randomized clinical trial. JAMA Ophthalmol. (2017) 135:1394–400. 10.1001/jamaophthalmol.2017.4970 29127432 PMC6583548

[21] Madi S Leon P Nahum Y D'Angelo S Giannaccare G Beltz J. Five-year outcomes of ultrathin Descemet stripping automated endothelial keratoplasty. Cornea. (2019) 3:1192–7. 10.1097/ICO.0000000000001999 31246680

[22] Suh LH Yoo SH Deobhakta A Donaldson KE Alfonso EC Culbertson WW. Complications of Descemet's stripping with automated endothelial keratoplasty: survey of 118 eyes at One Institute. Ophthalmology. (2008) 11:1517–24. 10.1016/j.ophtha.2008.01.024 18378315

